# MYCN and helicases DDX17 and DDX5 have opposite effects on the production of readthrough-associated chimeric transcripts

**DOI:** 10.1007/s00018-026-06254-6

**Published:** 2026-05-28

**Authors:** Valentine Clerc, Khouaila Aouadi, Jessica Valat, Xavier Grand, Lou-Sahra Khourab, Alizée Duquet, Nicolas Fontrodona, Matéo Bazire, Nicolas Rama, Didier Auboeuf, Benjamin Gibert, Franck Mortreux, Cyril F. Bourgeois

**Affiliations:** 1 Equipe Labellisée Ligue Contre Le Cancer, Laboratoire de Biologie Et Modelisation de La Cellule, Ecole Normale Superieure de Lyon, CNRS, UMR 5239, Inserm, U1293, Universite Claude Bernard Lyon 1, 69007 Lyon, France; 2 Cancer Research Center of Lyon (CRCL), INSERM U1052, Université Claude-Bernard Lyon 1, Hepatology Institute of Lyon, 69008 Lyon, France; 3 Apoptosis, Cancer and Development Laboratory- Equipe Labellisée La Ligue, LabEx DEVweCAN, Institut Convergence PLAsCAN, Centre de Recherche en Cancerologie de Lyon, INSERM U1052-CNRS 5286, Université Claude Bernard Lyon 1, 69008 Lyon, France; 4 Gastroenterology and Technologies for Health, Centre de Recherche en Cancerologie de Lyon, INSERM U1052-CNRS5286, Université Lyon 1, 69008 Lyon, France

## Abstract

DEAD box helicases DDX17 and DDX5 control transcription termination and the associated processing of the 3' end of pre-messenger RNAs. Here, we demonstrate that the transcriptional readthrough induced by DDX17 depletion, either alone or in combination with DDX5, leads to an increased production of chimeric transcripts from tandemly oriented gene, or tracRNAs, in neuroblastoma cells. Analysis of neuroblastoma tumours in which tracRNAs are abundant revealed that low expression of *DDX17* and *DDX5* genes is associated with high-risk tumours and poor overall patient survival, and inversely linked with *MYCN* oncogene amplification. We demonstrate that changes in MYCN expression do not affect the expression of either helicase, but alter transcription termination leading to the production of tracRNAs. MYCN acts on termination through its direct binding to the 3' region of genes and it interacts with DDX17, suggesting that it may interfere with the activity of the helicase. Collectively, our work reveals a novel function of MYCN in transcription termination and suggests that the deregulation of *MYCN* and *DDX17/DDX5* expression in neuroblastoma may lead to the expression of non-canonical and potentially harmful RNA molecules.

**Supplementary Information:**

The online version contains supplementary material available at 10.1007/s00018-026-06254-6.

## Introduction

In eukaryotes, the co-transcriptional processing of nascent RNAs produced by RNA polymerase II (RNAPII), *i.e.* 5' capping, splicing, 3' end cleavage and addition of the poly-A tail, is regulated not only by RNA binding proteins, but also by transcription and chromatin-associated factors [1, 2]. Transcription factors impact RNA processing in multiple manners, for example by regulating RNAPII elongation speed, interacting with RNA binding proteins or directly with RNA [3]. Yet, we know only a few examples of transcription factors that regulate alternative splicing by binding to chromatin sites near regulated RNA processing events rather than to the promoter [4, 5].

The tight connection between transcription termination and 3' end RNA processing is archetypal of this coordination of events. The slow down and release of RNAPII after the transcription of the polyadenylation site (PAS) and the cleavage of the nascent transcript are mutually dependent events, in a complex interplay involving factors acting on chromatin, at the level of the RNAPII complex, and/or on the RNA molecule [6–8]. Altering the expression or activity of these factors results in readthrough transcription past the TTS (transcription termination site) and in the extension of the 3' extremity of the transcript. Readthrough transcription has been observed in cells exposed to various stresses or viral infection, as well as in cancer [9–17], as recently reviewed [18, 19]. However, a recent survey of transcriptomic data revealed a large prevalence of readthrough transcription in healthy cells, affecting up to 34% of expressed protein-coding genes, suggesting a potential regulatory role of this phenomenon [20].

In some cases, readthrough transcription invades the downstream gene on the same DNA strand, generating *cis*-spliced transcripts containing exons from both genes. These RNA molecules have been called transcription-induced chimeras [21, 22], tandem RNA chimeras [23], products of conjoined genes [24, 25], readthrough fusions [26] or *cis*-splicing products of adjacent genes [27, 28]. Hereafter we propose to define them as tracRNAs for transcription readthrough-associated chimeric RNAs, to distinguish them clearly from mRNAs resulting from chromosomal rearrangements. As readthrough transcripts, tracRNAs are found in normal tissues [22, 29] and their production is considered as a possible mechanism to increase protein diversity since some of them can retain a functional open reading frame (ORF). Chimeric proteins produced from tracRNAs have been identified in cancer cells [26, 30–32]. Accordingly, tracRNAs have been identified in many types of cancers and associated with oncogenesis [33, 34].

Several splicing and/or polyadenylation factors impact the formation of tracRNAs [35], and chemical splicing inhibition represses their formation in neuroblastoma, a cancer in which most RNA fusions are intrachromosomal and correspond to tracRNAs [36]. The formation of tracRNAs can be associated to splicing defects in the adjacent invaded gene, highlighting the coordination of events that ensures the autonomous expression of neighbouring genes [37, 38]. Production of tracRNAs also involves chromatin-associated factors, such as the histone methyltransferase SETD2 [10] or CTCF [27, 28]. Recent reports further supported a function of CTCF in controlling PAS choice and transcription termination [39, 40].

Among the factors involved in 3' end processing of RNAPII transcript are DEAD box ATPases DDX17 and DDX5, two closely related proteins that have a variety of functions in gene expression, especially in transcription and RNA processing [41, 42]. Thanks to their helicase activity, these proteins modulate local DNA and RNA secondary structures and control the dynamics of transcription and RNA processing, impacting promoter and exon choice [43–49]. We and others have shown that the knockdown of DDX17 and DDX5, or their yeast ortholog Dbp2, also leads to readthrough transcription [40, 47, 48, 50, 51].

We show hereafter that the readthrough transcription induced by *DDX17* and *DDX5* silencing in neuroblastoma cells results in the production of several hundreds of tracRNAs, a fraction of which is also found in neuroblastoma tumours. Interestingly, we found that high *DDX17* and *DDX5* expression correlates with a good survival of neuroblastoma patients but that it is inversely correlated to the amplification of *MYCN*, a driving oncogene in this cancer [52]. Like the closely related transcription factor MYC, MYCN binds to most active promoters and regulates several aspects of RNAPII activity [53], but it also binds to many intragenic and intergenic sites. Here, we present evidence that MYCN directly controls transcription termination through its binding near the TTS of DDX17/DDX5-regulated genes. This new function suggests that the amplification of *MYCN*, combined to the suboptimal expression of *DDX17* and *DDX5*, could contribute to the increased expression of tracRNAs in high-risk neuroblastomas.

## Results

### DDX17 and DDX5 depletion enhances the expression of tracRNAs and chimeric proteins

**Fig. 1:**
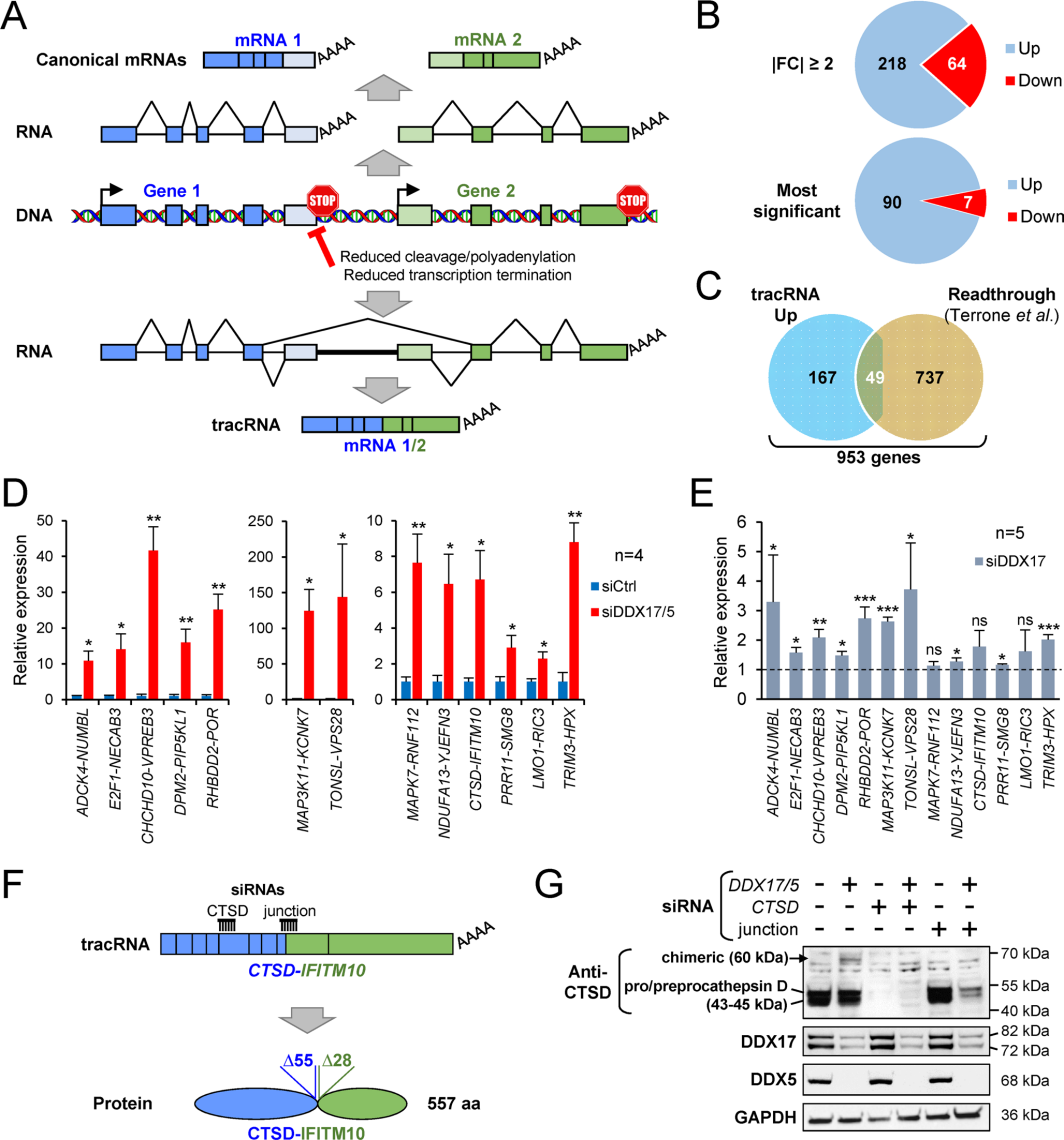
DDX17 and DDX5 depletion enhances the expression of tracRNAs. **A** Schematic representation of the production of readthrough induced tracRNAs. **B** Diagram showing the number of up and downregulated tracRNAs displaying at least a fold change of 2 and the most significant transcripts (*P*-val < 0.1). **C** Venn diagram showing the overlap between genes producing upregulated tracRNAs and the genes previously identified as displaying readthrough transcription. The list of 953 genes corresponds to the genes on which we carried out global analyses (Supp. Table 1). Note that within the 218 upregulated events from panel B, there are cases of tracRNAs that display a splicing between one exon from the same gene of origin to exons from different target genes, each time generating 2 different chimeras. As these events can be counted only once in panel C, this explains the total of 216 upregulated tracRNAs. **D** Quantification of tracRNAs upon silencing of both *DDX17* and *DDX5*. Expression of tracRNAs was monitored by RT-qPCR using primers spanning the chimeric junction and then normalized on the expression of the 5’ parental gene. Two-tailed paired t-test (* *P*-val < 0.05; ** *P*-val < 0.01). **E** Quantification of tracRNAs upon silencing of *DDX17*. Details are as in panel D. **F** Schematic representation of *CTSD-IFITM10* tracRNAs and putative chimeric protein. The sites targeted by the siRNAs used to validate the specificity of the band corresponding to CTSD-IFITM10 protein are indicated. **G** Western-blot showing the expression of the chimeric CTSD-IFITM10 protein and the canonical protein CTSD, as well as the validation of *DDX5* and *DDX17* silencing

We recently described a genome-wide effect of DDX17/DDX5 depletion on transcription termination in neuroblastoma cells SH-SY5Y cells [40]. Beside the numerous examples of transcriptional readthrough induced by DDX17/DDX5 depletion, our RNA-seq results also displayed splicing junction reads across adjacent genes. Typically, splicing skipped the terminal exon of the altered gene and linked its penultimate exon to the second exon of the following gene, generating a chimeric mRNA molecule or tracRNA (Fig. 1A). However, we also frequently observed complex alternative splicing patterns across exons from both genes. In some interesting cases chimeric junctions linked more than 2 adjacent genes, suggesting that DDX17/DDX5 depletion could induce the formation of multimeric mRNA molecules (*NTRK1-PEAR1-LRRC71* and *NDUFA13-YJEFN3-CILP2*).

We used the Arriba algorithm [54] to evaluate the number of tracRNAs whose expression is modified upon DDX17/DDX5 depletion. We identified 282 tracRNAs, 218 of which (77%) were upregulated and 64 (23%) downregulated (Fig. 1B, top panel, Supp. Table 1). Applying a more stringent selection threshold confirmed that the depletion of both helicases mostly increased the expression of tracRNAs (Fig. 1B, bottom panel). Hereafter we focused only on induced tracRNAs, and found that more than 22% of the genes displaying this pattern had been previously identified as genes with readthrough induced by DDX17/DDX5 depletion (Fig. 1C) [40]. Taken collectively, this allowed us to define a list of 953 genes whose termination is positively controlled by DDX17 and DDX5 (Supp. Table 1).

We next validated experimentally the expression of a selection of tracRNAs. First, we carried out RT-PCR assays using a primer overlapping the predicted RNA fusion between both genes, to specifically detect the tracRNA, that we systematically compared to the canonically produced mRNA. We observed an increased amount of all tested tracRNAs upon DDX17/DDX5 depletion, which did not systematically match with a change of expression of the corresponding canonical mRNA. Using RT-qPCR for a more precise quantification of a larger panel of tracRNAs, we showed that the expression of each tracRNA was increased between 2 and up to 150-fold in absence of DDX17/DDX5, compared to the canonical mRNA (Fig. 1D). Note that validated *CTSD-IFITM10*, *NDUFA13-YJEFN3* and *TRIM3-HPX* tracRNAs were not predicted as significant events by Arriba but were identified while browsing through our RNA-seq data. This suggested that the number of tracRNAs induced by DDX17/DDX5 depletion is underestimated.

We also tested the effect of single *DDX17* knockdown on the production of previously validated tracRNAs. We observed an increased expression of all tracRNAs, even though the effect was less pronounced than in the double knockdown condition (Fig. 1E). This experiment showed that the depletion of only one of the two helicases is sufficient to induce readthrough transcription.

Some tracRNAs maintain the open reading frame of their respective canonical transcripts and escape degradation by the nonsense-mediated mRNA decay pathway. For example, the *CTSD-IFITM10* tracRNA is cleaved and polyadenylated at the canonical PAS of the *IFITM10* gene. This chimeric mRNA is predicted to produce a chimeric protein of 557 amino acids (60 kDa), lacking 55 and 28 amino acids from the C-terminal part of CTSD and N-terminal part of IFITM10 proteins, respectively (Fig. 1F). This chimeric protein was identified in breast cancer cells [26], and we sought to detect it in neuroblastoma cells, using an antibody against Cathepsin D. We identified expected proteins of 43 and 45 kDa, but also a discrete 60 kDa band that was detected only upon depletion of DDX17 and DDX5 (Fig. 1G). We estimated that its relative expression had increased by at least fivefold compared to the control condition. All bands nearly disappeared when cells were treated with a siRNA targeting the body of the *CTSD* transcript. Furthermore, treating cells with another siRNA specifically targeting the *CTSD-IFITM10* junction also led to a complete loss of the 60 kDa band. Note that this treatment also reduced the level of canonical Cathepsin D, likely because of a non-specific effect of the junction siRNA on canonical mRNAs. This result demonstrated that the siDDX17/DDX5-induced tracRNAs can produce chimeric proteins, which could have a strong functional impact in cells.

### The expression of the DDX17 gene is reduced in high-risk neuroblastomas

**Fig. 2:**
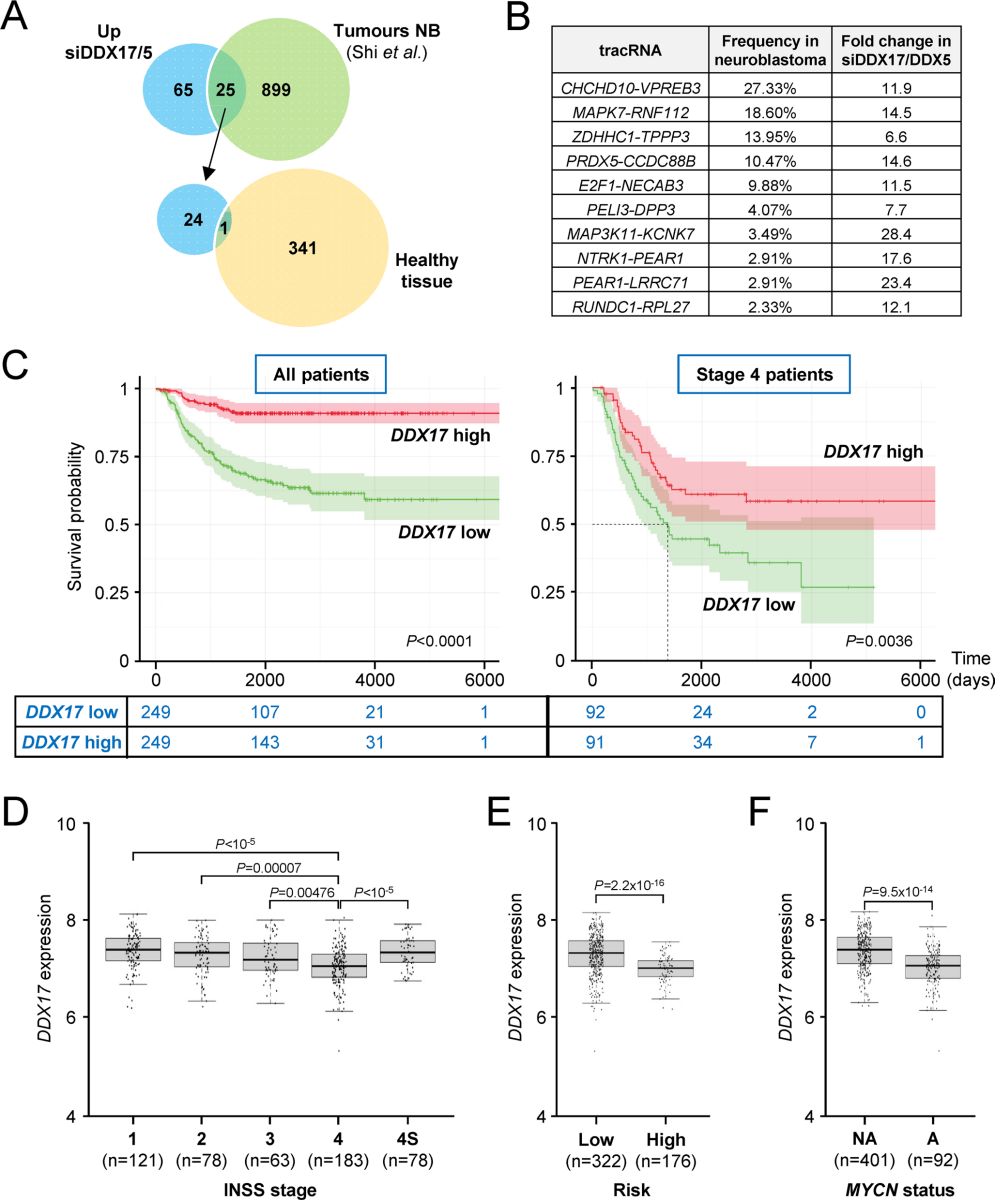
The expression of the *DDX17* gene is reduced in high-risk neuroblastomas. **A** Venn diagrams showing the number of tracRNAs (defined as in Fig. 1A) that are upregulated upon DDX17/DDX5 depletion and identified in neuroblastoma tumors. **B** Top 10 of tracRNAs identified both in neuroblastoma tumours and upregulated upon DDX17/DDX5 depletion. **C** Kaplan–Meier curves showing the overall survival of neuroblastoma patients, separated in 2 groups of high and low *DDX17* expression (relative to the median value of the group). Left diagram: all patients (*n* = 498). Right diagram: stage 4 patients (*n* = 183). The number of patients in each group and for each time point is indicated below. **D** Box plot of *DDX17* expression relative to tumor stage (INSS classification). ANOVA corrected for multiple comparisons with Tukey’s tests. Only the comparison between Stage 4 and other stages is shown, the other comparisons were not significant. **E** Box plot of *DDX17* expression relative to low/high risk classification. Two-tailed *t*-test. **F** Box plot of *DDX17* expression relative to amplified (A) or non-amplified (NA) *MYCN* status. Two-tailed *t*-test

Recently, neuroblastoma tumours were shown to express large amounts of tracRNAs [36], which prompted us to look whether DDX17/DDX5-regulated chimeras can be found in these tumours. Of the 90 most significantly induced tracRNAs from our analysis, 25 were also identified in neuroblastomas (Fig. 2A, top panel, Supp. Table 1). Of note, 4 of these 25 tracRNAs were detected in at least 10% of neuroblastomas (Fig. 2B), whereas only 1 of them (*SLC29A1-HSP90AB1*) was also detected in a control cohort of 161 healthy adrenal gland samples (Fig. 2A, bottom panel).

Our earlier work showed that DDX17 and DDX5 are involved in early stages of retinoic acid-induced differentiation of neuroblastoma cells [46]. To explore further the link between the two helicases and this cancer, we analysed the expression of their genes in the clinically annotated SEQC neuroblastoma cohort [55]. We found that a high expression of *DDX17* was significantly correlated with a better survival probability of patients (Fig. 2C, left panel). This was true also when the Kaplan-Meyer analysis was performed only on the most aggressive stage 4 patients (International Neuroblastoma Staging System INSS) (Fig. 2C, right panel). More specifically, *DDX17* expression is significantly lower in stage 4 tumours than in any other neuroblastoma stage (Fig. 2D), and is also significantly reduced in high-risk tumours (Fig. 2E). Finally, we found a negative association between *DDX17* expression and the amplification of the *MYCN* oncogene, which is a well-established driver and marker of poor prognostic in neuroblastoma [52] (Fig. 2F). For the *DDX5* gene, results were more contrasted but as for *DDX17*, a low *DDX5* expression was correlated with a lower survival probability and with *MYCN* amplification. This is in contrast with previous findings that associated a high expression DDX5 with an unfavourable outcome in neuroblastoma patients [56].

In conclusion, our results established a link between the expression of *DDX17*, and to a lower extent of *DDX5*, and the severity of neuroblastoma. This raised the interesting possibility that a suboptimal expression of DDX17 and/or DDX5, which leads to transcription termination defects of hundreds of genes in cultured cells, could promote the abnormal expression of a subset of tracRNAs in aggressive neuroblastomas.

### Overexpression of MYCN increases transcriptional readthrough and tracRNA formation

The negative link between the amplification of *MYCN* and a lower expression of both *DDX17* and *DDX5* genes suggested that MYCN could control the expression of both helicases. To test this hypothesis, we used two different neuroblastoma cell lines with and without an amplification of *MYCN* to modulate the expression of the oncogene. As c-MYC was recently shown to regulate readthrough transcription [57], we also investigated whether variations in MYCN expression could affect transcription termination in neuroblastoma cells, using a subset of previously validated DDX17/DDX5-regulated tracRNAs (Fig. 1) and readthrough events [40] as readouts.

**Fig. 3:**
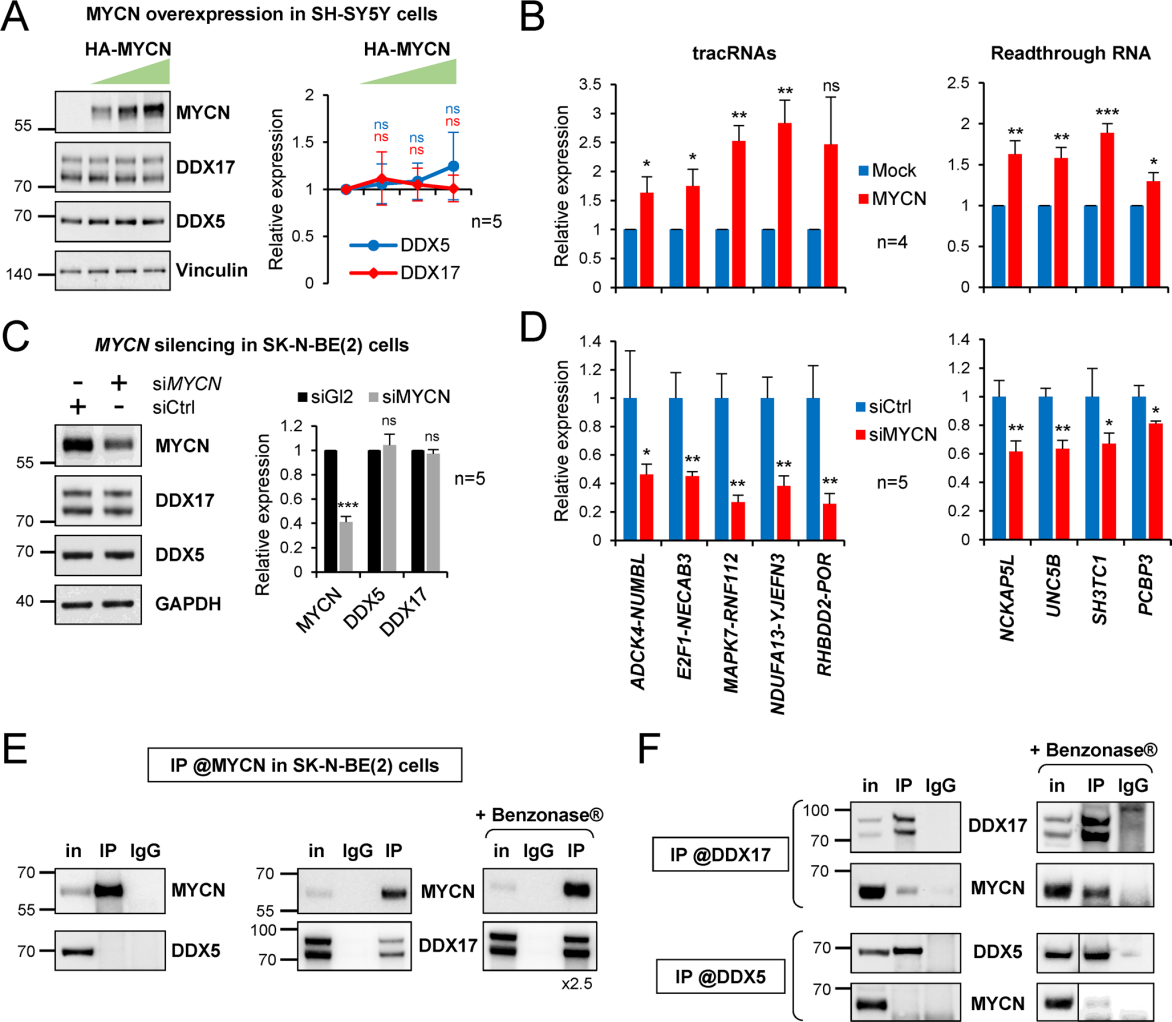
MYCN enhances the formation of tracRNAs and interacts with DDX17. **A** Western-blot showing DDX17 and DDX5 protein levels upon increasing expression of MYCN in SH-SY5Y cells. Quantification of the western-blot (normalised to vinculin) is shown on the right. **B** Expression of tracRNAs and readthrough transcripts upon MYCN overexpression. Details are as in Fig. 1D. Ratio paired *t*-test (*P-val < 0.05; **P-val < 0.01; ***P-val < 0.001). **C** Western-blot showing DDX17 and DDX5 protein levels upon MYCN depletion in SK-N-BE(2) cells. Quantification of the western-blot (normalised to GAPDH) is shown on the right. **D** Expression of tracRNAs and readthrough transcripts upon MYCN depletion. Unpaired Mann–Whitney test (*P-val < 0.05; **P-val < 0.01). **E** Immunoprecipitation assays of endogenous MYCN in SK-N-BE(2) cells to detect its association with DDX5 and DDX17. The cell lysate was pre-treated or not with Benzonase®, as indicated, to remove all nucleic acids. **F** Immunoprecipitation of endogenous DDX5 and DDX17 in SK-N-BE(2) cells to detect their association with MYCN, in the presence or absence of Benzonase®

First, we transfected SH-SY5Y cells (no *MYCN* amplification) with increasing amounts of an HA-tagged MYCN-encoding plasmid. MYCN protein was barely detectable in control cells and strongly induced upon transfection, but this did not result in any significant change in DDX17 and DDX5 protein levels (Fig. 3A). However, under these conditions we observed an increased readthrough transcription and production of tracRNAs (Fig. 3B).

In parallel, we downregulated MYCN expression in the *MYCN*-amplified SK-N-BE(2) cell line, using specific siRNAs which reduced MYCN protein level by nearly 60%. This depletion did not affect the levels of either DDX17 or DDX5 protein (Fig. 3C), but it significantly reduced readthrough transcription and tracRNA expression (Fig. 3D). Importantly, steady-state levels of the corresponding canonical transcripts remained unaffected by variations in MYCN expression, suggesting that MYCN altered transcription termination and as a consequence, tracRNA production, in a manner that is distinct from its promoter-associated activity.

### MYCN interacts with DDX17

Since the effect of MYCN on transcription termination is the opposite of that of DDX17 and DDX5, we hypothesised that MYCN could in some way interfere with the function of helicases at the 3' end of their target genes. Thus, we tested whether MYCN could interact with these helicases by carrying out co-immunoprecipitation (co-IP) assays in SK-N-BE(2) cells. As shown in Fig. 3E, endogenous MYCN and DDX17, but not DDX5, co-immunoprecipitated efficiently. The efficiency of MYCN IP was increased by 2.5-fold when the cell lysates were treated with Benzonase®, which similarly improved the detection of DDX17. This result excluded the contribution of nucleic acids and suggested a direct interaction between the two proteins. Next, we carried out the reverse experiment and immunoprecipitated DDX17 and DDX5 from SK-N-BE(2) cell lysates. In absence of Benzonase®, endogenous MYCN was detected only in the DDX17 IP, and not in the DDX5 IP (Fig. 3F), confirming the results of MYCN IPs. Benzonase® treatment improved the detection of MYCN in DDX17 IP samples, and it also allowed to detect a weak amount of MYCN protein in the DDX5 IP. Altogether, these results revealed the existence of a complex between MYCN and DDX17 in neuroblastoma cells, and they underlined the specificity of this interaction, which could alter the activity of DDX17 and lead to enhanced readthrough transcription.

### MYCN binding is enriched near the TTS of DDX17/DDX5-regulated genes

**Fig. 4:**
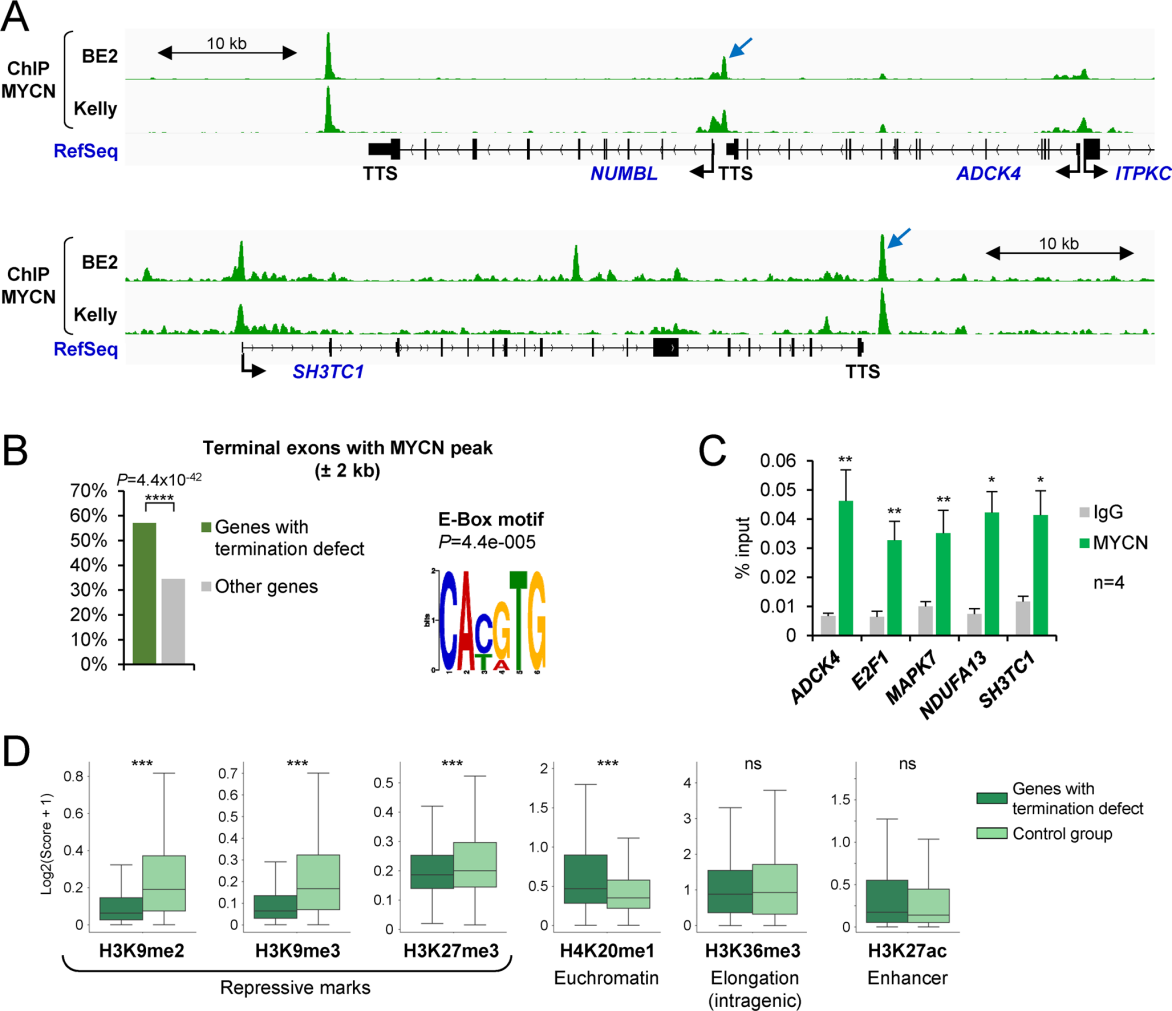
MYCN binds near the TTS of DDX17/DDX5-regulated genes. **A** Typical examples of DDX17/DDX5-regulated genes displaying MYCN binding near their 3’ end. **B** Left panel: genome wide analysis of MYCN binding near terminal exons of DDX17/DDX5-regulated genes and control genes in neuroblastoma cell lines. Tukey’s test. Right panel: MEME analysis showing the enrichment of the E-Box motif within MYCN binding sites. **C** ChIP-qPCR analysis of MYCN binding near the 3’ end of DDX17/DDX5-regulated genes. Paired *t*-test (*P-val < 0.05; **P-val < 0.01). **D** Analysis of histone mark enrichment near the termination region of DDX17/DDX5-regulated genes (TTS −300 to + 1500 bp) and control genes in SK-N-SH cells. Two-sided Wilcoxon rank-sum test (***P < 0.001; ns, not significant)

To test the hypothesis of a direct effect of MYCN on transcription termination, we re-analysed previously published ChIP-seq datasets performed in several neuroblastoma cell lines [58–60]. We found that the 3' region of genes displaying transcriptional readthrough or tracRNA production upon *DDX17/DDX5* knock-down or MYCN overexpression often exhibited at least one MYCN peak, detected across several cell lines (Fig. 4A). A global analysis of all DDX17/DDX5-regulated genes confirmed this observation, as the chromatin region overlapping their terminal exon was significantly enriched in MYCN binding sites, compared to non-regulated genes (Fig. 4B). These binding sites are enriched in the motif corresponding to the classical E-Box recognised by members of the Myc family (Fig. 4B). The binding of MYCN near the 3' end of our model genes was also validated by ChIP-qPCR assays in SK-N-BE(2) cells (Fig. 4C). Together with results of Fig. 3 showing that MYCN does not affect DDX17 and DDX5 expression, these experiments strongly suggested that the effect of MYCN on transcription termination is direct.

To extend our analysis of the epigenetic state of those regulated genes, we also examined the enrichment of several histone marks downstream of their TTS, using ENCODE [61] ChIP-seq datasets from SK-N-SH neuroblastoma cells. This revealed a reduction in repressive marks (H3K9me2/3 and H3K27me3), alongside an increase in a mark associated with open and active chromatin (H4K20me1) (Fig. 4D). No difference was observed for marks associated with active intragenic transcription (H3K36me3) and active enhancers (H3K27ac) (Fig. 4D). These data suggest that the chromatin downstream of genes regulated by DDX17/DDX5 is in a permissive state that could promote readthrough transcription when their regulation is altered.

### The forced recruitment of MYCN near the termination region induces the production of tracRNAs

**Fig. 5:**
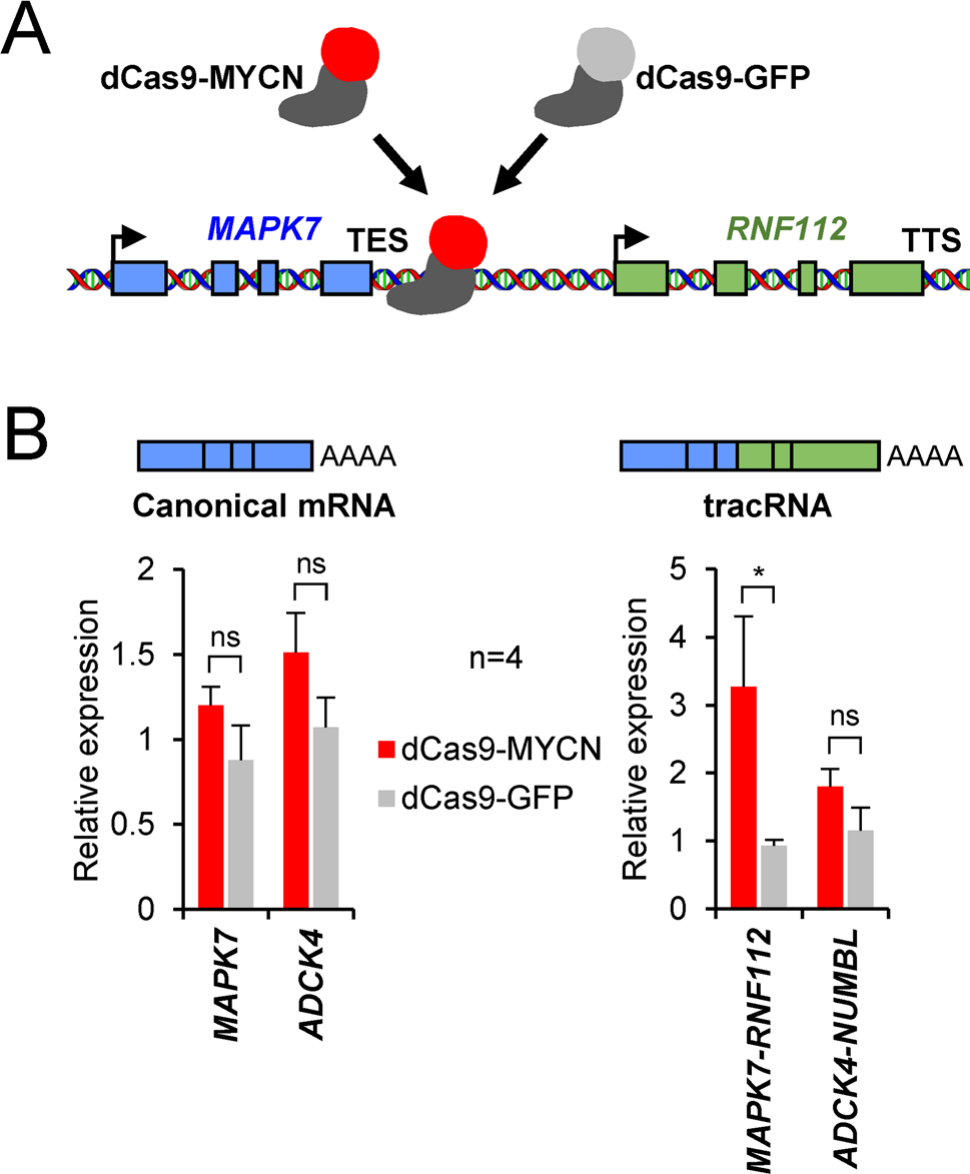
The forced recruitment of MYCN near the termination region is sufficient to induce the formation of tracRNAs. **A** Strategy for the recruitment of MYCN (fused to a catalytically inactive dCas9) at the 3’ end of the *MAPK7* gene. **B** Quantification of canonical *MAPK7* transcript and *MAPK7-RNF112* tracRNA in cells transfected with dCas9-MYCN or dCas9-GFP, relative to transfection without specific gRNA. Paired *t*-test (*P-val < 0.05; **P-val < 0.01)

Finally, to evaluate the consequence of MYCN binding near the TTS on transcriptional readthrough and tracRNA production, we designed an experiment in SH-SY5Y cells (low endogenous MYCN level) in which we could force the recruitment of MYCN downstream of the TTS of the *MAPK7* gene. For this, we co-transfected cells with two plasmids, one expressing the MYCN protein fused to a catalytically inactive "dead" Cas9 protein and the other expressing specific RNA guides to target dCas9-MYCN downstream of the *MAPK7* gene (Fig. 5A). A plasmid expressing dCas9-GFP fusion protein was used as a control to rule out a possible non-specific action of the dCas9 fusion protein on transcription through steric hindrance. We verified by ChIP-qPCR that fusion proteins were indeed bound at their target site upon transfection of RNA guides.

We then quantified the expression of the *MAPK7* gene, which remained unaffected by the recruitment of either dCas9-MYCN or dCas9-GFP, as did the control *ADCK4* gene (Fig. 5B, left panel). In contrast, the expression of the *MAPK7-RNF112* tracRNA was enhanced specifically upon the binding of the dCas9-MYCN between the two genes, while the *ADCK4-NUMBL* tracRNA remained unchanged (Fig. 5B, right panel). This experiment demonstrated that the binding of MYCN near the TTS is sufficient to inhibit the normal termination process and promote the formation of a tracRNA.

## Discussion

In this study, we showed that MYCN, a transcription factor with a major oncogenic effect in many cancers, in particular neuroblastoma, promotes readthrough transcription and the production of tracRNAs. This new function of the oncoprotein is dependent on its binding to chromatin in the TTS region. Importantly, MYC was also recently described to bind near the TTS and to be associated with altered readthrough transcription [57], which underlines the conserved nature of this function within the MYC protein family. Our study is a new illustration of moonlighting functions that a transcription factor can have upon its binding to non-promoter sites, such as the effects of intragenic binding of transcription factors on alternative splicing [4, 5].

Highlighting the features associated with MYCN/MYC binding at the TTS of genes helps to understand how these transcription factors control termination. First, in line with the lack of enrichment of the typical enhancer mark H3K27ac in our analysis, Wang and colleagues distinguished MYC-associated TTS from classical transcription enhancers, especially because of their location and because of the diversity of their effect [57]. Indeed, they identified examples of increased or decreased readthrough transcription of genes associated to MYC binding. This differs from our results showing a repressed termination of each tested gene by MYCN, but we cannot exclude that the limited size of our panel does not reflect the complete activity of MYCN.

MYC/MYCN could prevent termination at the end of the gene by activating RNAPII, as they are both known to induce productive elongation [62–64]. MYC proteins are known to interact with multiple chromatin remodeling complexes [53], whose ectopic recruitment to the 3’ end of gene may alter the local epigenetic environment, affect RNAPII kinetics and interactome and disturb the chain of events leading to termination, as proposed for MYC [57]. Interestingly, our analysis of histone marks in an untreated neuroblastoma cell line (no depletion of DDX17/DDX5 and no *MYCN* amplification) indicates that the readthrough-associated chromatin of DDX17/DDX5-regulated genes is in a more open and transcriptionally permissive state, compared to unregulated genes. This particular epigenetic context may be more disposed to alterations leading to readthrough transcription beyond the TTS.

In our study, one common feature of MYCN-altered genes is that their termination depends on helicases DDX17 and DDX5. We showed previously that DDX17/DDX5-regulated genes display a looped 3D conformation that brings closer their promoter and TTS [40]. Interestingly, this specific 3D organisation, which we showed to be critical for the proper selection of the polyadenylation/termination site [40], is also a key aspect of MYC-associated TTS [57]. MYC/MYCN could therefore contribute to the crosstalk between the promoter and factors involved in 3' end processing and termination, like many other factors [7, 65]. These findings reinforce the concept of a tight spatial coordination between initiation and termination of transcription [40, 66].

The fact that MYCN binds preferentially downstream of DDX17/DDX5-regulated genes, and that it efficiently co-immunoprecipitates with DDX17, both strongly advocate for a functional interplay between the transcription factor and the helicase, at least in neuroblastoma cells. One hypothesis is that, when overexpressed, MYCN may interfere with normal DDX17 interactions with its protein partners, including its heterodimerisation partner DDX5. Alternatively, it may interfere with DDX17 binding to chromatin or RNA, or with DDX17 activity on DNA or RNA structures. Consequently, this may impact DDX17/DDX5-mediated 3D gene looping [40] and termination. Future work will help to determine which of those non-mutually exclusive hypotheses is correct, or if another mechanism is involved.

The readthrough transcription induced either by depletion of DDX17, alone or with DDX5, or by MYCN overexpression, leads to the production of tracRNAs in neuroblastoma cells. Among the most significantly induced tracRNAs, 27% were previously found in neuroblastoma tumors and not in non-tumoural adrenal gland tissue [36]. This suggests that this subset of chimeric RNA molecules could potentially be used as biomarkers for this cancer, but further studies will be required to determine their level of specificity, as tracRNAs have been identified in many types of cancers [33, 34].

If production of tracRNA in healthy cells was proposed to increase protein diversity [22], their abnormal expression in cancer cells can have several consequences [67]. First, a fraction of tracRNAs can retain a functional ORF allowing the translation of chimeric or fusion proteins [26, 30–32], and we validated the production of CTSD-IFITM10 protein upon DDX17/DDX5 depletion. In the future, an important objective will be to study the effects of MYCN and search for such chimeric proteins in neuroblastoma cell proteomes. Chimeric proteins can generate highly immunogenic neo-antigens, which may be unique to the tumour and could be targeted by the immune system, raising new hopes for cancer therapies [68]. Chimeric proteins produced from tracRNAs could also alter the normal protein interaction network and activate cell growth pathways by acting as oncoproteins, or alter the cell response to antitumour treatments. Second, chimeric transcripts can act as functional long noncoding RNAs (lncRNA). For example, the *SLC45A3-ELK4* tracRNA acts as lncRNA and regulates cell growth in prostate cancer, where its higher expression level has been associated with disease progression and metastasis [69, 70]. Future analyses will determine whether the tracRNAs we have identified have protumoral properties or can influence tumour response to treatment.

Our results show that a reduced expression of *DDX17* or *DDX5* genes is significantly associated with a higher risk and shorter patient survival in neuroblastoma patients. These results differ from a previous report showing a link between high *DDX5* expression and poor survival of neuroblastoma patients [56], but we used a much larger cohort of tumours (*n* = 498 versus *n* = 42). Importantly, the fact that high expression of *DDX17* is associated with a better survival of stage 4 patients reduces the possibility that it is directly and solely linked to *MYCN* amplification, which is a less predominant risk factor in this class of tumours. We will have to confirm these results at the protein level and to investigate the possible contribution of both helicases to the mechanism of oncogenesis. Based on our previous [40] and present results, we propose that a combined *MYCN* overexpression and suboptimal expression of *DDX17* and/or *DDX5* may create unfavourable conditions leading to deep transcriptome alterations in neuroblastoma cells, especially readthrough transcription and production of tracRNAs. Understanding better the interplay between these factors will be of great importance to appreciate their impact on tumorigenesis.

Finally, showing that *DDX17* knockdown alone is sufficient to induce readthrough and tracRNA production is interesting in the context of the recent association of de novo heterozygous *DDX17* variants with neurodevelopmental disorders [71]. The reduced expression and/or activity of DDX17 in those patients may induce termination defects that may contribute to their altered neuronal development, an hypothesis that will be tested in future studies.

## Materials and methods

### Plasmids and cell culture

Human SH-SY5Y and SK-N-BE(2) cells (ECACC) were grown and transfected essentially as described previously [46]. For standard *DDX17/DDX5* silencing experiments, 20 nM of siRNA (sequences in Supp. Table 2) were mixed with Lipofectamine™ RNAiMax (ThermoFisher Scientific) following the manufacturer’s instructions and cells were harvested 48 h after transfection. Note that for *DDX17* silencing, we used a mixture of 2 different siRNAs and a total concentration of 60 nM siRNAs. For MYCN overexpression, cells were plated in 6-well-plates to reach 70% confluence, and then transfected with 0.5 to 1 μg pCDNA3-HA-hMYCN (Addgene #74163) using JetPrime® (Polyplus Transfection) following the manufacturer’s instructions. Cells were harvested 48 h after transfection.

For dCas9-MYCN experiments, the hMYCN cDNA was subcloned into the dCas9-empty-GFP plasmid (a gift from Reini F. Luco, Institut Curie, Orsay, France). This cloning step was done by RD Biotech. We cloned the sequences corresponding to the two RNA guides into the *Bsm*BI site of the CRIZI plasmid (provided by Philippe Mangeot, CIRI, Lyon, France). SH-SY5Y cells were plated in 6-well-plates to reach 70% confluence and transfected with 1 μg of dCas9-HA-MYCN plasmid and 1 μg of sgRNA-containing plasmid (500 ng of each guide, sequences in Supp. Table 2) using jetPRIME (PolyPlus Transfection).

### Western blot and co-immunoprecipitation

Protein extraction and western blotting were carried out as previously described [45]. Primary antibodies used for western-blotting: DDX5 (ab10261, Abcam), DDX17 (ab24601, Abcam), GAPDH (sc-32233, Santa Cruz Biotechnology), MYCN (sc-53993, Santa Cruz Biotechnology), CTSD (21327–1-AP, Proteintech). For co-immunoprecipitation, SK-N-BE(2) cells were harvested and gently lysed in IP buffer (20 mM Tris–HCl pH 7.5, 150 mM NaCl, 2 mM EDTA, 1% NP40, 10% glycerol and protease/phosphatase inhibitors) for 30 min at 4 °C under constant mixing. The nuclear lysate was centrifuged for 15 min to remove cell debris and soluble proteins were quantified by BCA (ThermoFisher). The lysate, treated or not with 250 U/ml Benzonase® (Merck-Millipore) for 30 min at 37 °C, was then split in aliquots of 1 mg of proteins. Each fraction received 4 μg of antibody and the incubation was left overnight at 4 °C under rotating mixing. The following antibodies were used for IP: rabbit anti-DDX17 (19910–1-AP, Proteintech) or control rabbit IgG (ThermoFisher), goat anti-DDX5 (ab10261, Abcam) or control goat IgG (Santa Cruz Biotechnology), and mouse anti-MYCN (sc-53993, Santa Cruz Biotechnology) or control mouse IgG. The next day, the different lysate/antibody mixtures were divided and incubated with 50 μg Dynabeads Protein G/A (ThermoFisher), for 4 h at 4 °C under rotating mixing. Beads were washed 5 times with IP buffer, elution was performed by boiling for 5 min in SDS-PAGE loading buffer prior to analysis by western-blotting.

### RNA extraction and real-time quantitative PCR

Total RNAs were isolated using TriPure Isolation Reagent (Roche). For reverse transcription, 2 μg of purified RNAs were treated with Dnase I (ThermoFisher) and retrotranscribed using Maxima reverse transcriptase (ThermoFisher), as recommended by the supplier. Potential genomic DNA contamination was systematically checked by performing negative RT controls in the absence of enzyme and by including controls with water instead of cDNA in qPCR assays. PCR reactions were carried out described previously [46]. For qPCR analyses, the specificity and linear efficiency of each primer set (sequences are in Supp. Table 2) was first verified by establishing a standard expression curve with various amounts of human genomic DNA or cDNA. qPCR reactions were carried out on 0.625 ng of cDNA using a LightCycler 480 System (Roche), with the SYBR® Premix Ex Taq (Tli RNaseH Plus, Takara), under conditions recommended by the manufacturer. Melting curves were controlled to rule out the existence of non-specific products. Relative DNA levels were calculated using the 2-ΔΔCt method (using the average Ct obtained from technical duplicates or triplicates) and were normalised to the expression of *GAPDH* RNA.

### Chromatin immunoprecipitation

A total of 10^7^ cells were crosslinked with 1% formaldehyde for 10 min at room temperature. Crosslinking was quenched by addition of 0.125 M glycine. Nuclei were isolated by sonication using a Covaris S220 (2 min, Peak Power: 75; Duty Factor: 2; Cycles/burst: 200), pelleted by centrifugation at 1000 g for 5 min at 4 °C, washed once with FL buffer (5 mM HEPES pH 8.0, 85 mM KCl, 0.5% NP40) and resuspended in 1 ml shearing buffer (10 mM Tris–HCl pH 8.0, 1 mM EDTA, 0.1% SDS). Chromatin was sheared in order to obtain fragments ranging from 200 to 800 bp using Covaris S220 (20 min, Peak Power: 140; Duty Factor: 5; Cycles/burst: 200). Chromatin was next immunoprecipitated overnight at 4 °C with 4 μg of mouse anti-MYCN antibody (sc53993, Santa Cruz Biotechnology), or an equivalent amount of the corresponding IgG Isotype control (ThermoFisher), and then incubated for 4 h with 30 μl of Dynabeads® Protein A/G (ThermoFisher). Complexes were washed with 4 different buffers: Low salt buffer (20 mM Tris–HCl pH 8.0, 150 mM NaCl, 1% Triton X-100, 0.1% SDS, 2 mM EDTA), High salt buffer (20 mM Tris–HCl pH 8.0, 500 mM NaCl, 1% Triton X-100, 0.1% SDS, 2 mM EDTA), Low LiCl buffer (10 mM Tris–HCl pH 8.0, 0.5 M LiCl, 1% NaDoc, 1% NP40), Tris/EDTA (10 mM Tris–HCl pH 8.0, 1 mM EDTA), and were eluted in Elution buffer (200 mM NaCl, 1% SDS, 0.1 M NaHCO_3_, 20 μg Proteinase K) overnight at 65 °C. The immunoprecipitated chromatin was purified by phenol–chloroform extraction and ethanol precipitation, and analysed by qPCR. Values were normalised to the signal obtained for the immunoprecipitation with control IgG.

### In silico prediction of chimeric RNAs from RNA-seq data

The raw RNA-seq data were described previously [40] and are accessible in the Gene Expression Omnibus repository (accession number GSE183205). Raw reads were pre-processed using fastp (v0.23.2) [72] and mapped to the human reference genome (hg19, GRCh37.87) using STAR (v2.7.8a) [73]. Mapped reads were filtered using samtools (v1.11) [74]. Gene fusions were detected using Arriba (v2.3.0) [54] and results were parsed using an R script. Split and discordant reads identified by Arriba were counted for each fusion and compared to control condition. Differentially fused genes (DFG) were analysed using the DESeq2 package (v1.40.1) [75]. The complete pipeline is available in Nextflow [76] at https://gitbio.ens-lyon.fr/LBMC/regards/nextflow/-/blob/master/src/arriba_fusion.nf.

### Statistical analysis of DDX17 and DDX5 expression in neuroblastoma tumours

To test the expression of *DDX17* and *DDX5* in neuroblastomas, we used the previously described cohort of 498 tumours (GEO accession number: GSE62564) [77], in which patients groups were defined according to the International Neuroblastoma Staging System (INSS). Survival curves were generated by the Kaplan–Meier method, and statistical analyses were carried out as described previously [78, 79].

### Analysis of MYCN ChIP-seq data

To analyse the relative proximity of DDX17/DDX5-regulated terminal exons to MYCN binding sites, we first generated a BED file containing a merged list of MYCN peaks identified in several neuroblastoma cell lines [58–60] (Supp. Table 3), re-analysed as follows. Raw reads were pre-processed using fastp (v0.23.2) [72] and mapped to the human reference genome (hg19, GRCh37.87) using bowtie2 (v2.5.2) [80]. Mapped reads were filtered using samtools (v1.11) [74] and then formatted using deepTools2 (v3.5.1) [81]. Peak calling analysis was carried out with MACS (v3.0.0a6) [82]. Nearest exons from peak summits were identified using to BEDtools (v2.25.0) [83]. The complete ChIP-seq pipeline is available in Nextflow [76] at https://gitbio.enslyon.fr/xgrand/ChIPster.

We next calculated the genomic distance (negative or positive for upstream and downstream peaks, respectively) between each exon from the FasterDB database [84] and the summit of the closest MYCN peak. Only genes having at least one internal exon were considered. We performed a logistic regression analysis to test if the 3' terminal exons regulated by DDX5/DDX17 (*n* = 933) are closer to MYCN peaks than unregulated terminal exons. (*n* = 18,482). We modeled the proximity to a MYCN peak according to the two groups of exons using the glm function, with family = binomial (‘logit’) in R software. A MYCN peak was considered as close to an exon if its center is located within an exon or within an interval of 1 base to 2 kb upstream or downstream of the exon. To test the differences between the two groups of exons, a Tukey’s test was used (with R, emmeans function from emmeans library). To search for enriched motifs within MYCN peaks, we used the MEME tool (version 5.4.1) from the MEME suite [85], with the defaulyt parameters. Starting from the summit of the nearest MYCN peak, we extended the sequence by 250 nucleotides on either side in order to define the coordinates of each peak.

### Analysis of histone marks

To study the enrichment of histone marks at the transcription termination site (TTS) of DDX17/DDX5-regulated genes, we generated a BED file containing the genomic coordinates of TTS regions, defined as 300 bp upstream to 1,500 bp downstream of the TTS, based on the human reference genome (hg19, GRCh37.8). Genes were divided into two groups: a group including genes that produce readthrough transcripts or tracRNAs upon DDX17/DDX5 depletion in SH-SY5Y cells (*n* = 702) and a control group of unaffected genes (*n* = 9806). Only genes with an average DESeq2-based basemean expression of at least 10 in our RNA-seq experiment were retained. In addition, to avoid including promoter-associated signals in our analysis, we only kept genes whose nearest downstream protein-coding or long non-coding RNA (lncRNA) gene was located at least 3 kb from the TTS. We retrieved processed ChIP-seq bigWig files for several histone marks of interest (Supp. Table 3) from the ENCODE portal [61]. These datasets were derived from untreated SK-N-SH cells, the parental cell line of SH-SY5Y. We next calculated the mean histone mark enrichment score for each TTS region using deepTools2 (v3.5.6) [81]. We performed statistical analyses using Python (v3.12.3). Mean enrichment scores were transformed using the Log2(Score + 1) function. To test the differences in histone mark enrichment between the two groups of genes, a two-sided Wilcoxon rank-sum test was used (with Python, mannwhitneyu function from the scipy.stats librairy).

## Supplementary Information

Below is the link to the electronic supplementary material.

Supplementary file1 (XLSX 51 KB)

Supplementary file2 (XLSX 13 KB)

Supplementary file3 (XLSX 12 KB)

Supplementary file4 (PDF 936 KB)

## Data Availability

Raw RNA-seq data are accessible in the Gene Expression Omnibus repository (accession number GSE183205). Cohort of 498 neuroblastoma tumours: GEO accession number: GSE62564. Public data used for MYCN and histone marks ChIP-seq analyses are listed in Supp. Table 3.
