# Supplementary material for: MYCN and helicases DDX17 and DDX5 have opposite effects on the production of readthrough-associated chimeric transcripts
Source: Cell Mol Life Sci. 2026 May 28;83(1):290. doi: 10.1007/s00018-026-06254-6 (PMC13429570; doi:10.1007/s00018-026-06254-6)

Supplementary information

MYCN and helicases DDX17 and DDX5 have opposite effects on the production of readthrough-associated chimeric transcripts

Valentine Clerc, Khouaila Aouadi, Jessica Valat, Xavier Grand, Lou-Sahra Khourab, Alizée Duquet, Nicolas Fontrodona, Matéo Bazire, Nicolas Rama, Didier Auboeuf, Benjamin Gibert, Franck Mortreux, Cyril F. Bourgeois

Legends for Supplementary Figures

Supp. Figure 1. Typical examples of tracRNAs induced by DDX17 and DDX5 depletion. **A.** Screenshot from the Integrative Genome Viewer (IGV) browser showing the RNA-seq reads aligning to *CTSD* and *IFITM10* genes. The sequencing coverage of control (blue) and siDDX17/DDX5 (red) conditions shows a reduction of the steady-state expression the *CTSD* gene, yet the number of reads spanning the junction between exons of both genes is higher upon DDX17/DDX5 depletion. Most reads characterising the *CTSD-IFITM10* tracRNAs link the one-to-the-last exon of *CTSD* to the second exon of *IFITM10*. **B.** Other IGV screenshots, in which only the sequencing coverage and splicing junctions are shown. Control and siDDX17/DDX5 conditions are in red and blue, respectively.

Supp. Figure 2. A. Schematic representation of canonical mRNAs and tracRNA, which indicates the position of the primers designed to quantify their respective level (red arrows). **B.** Validation of the expression of tracRNAs by RT-PCR. Expression of tracRNAs was monitored using primers spanning the chimeric junction and compared to the expression of the parental gene. Controls for reverse transcription (RNA in the RT) and PCR (no cDNA) are included. The asterisk on the *GAPDH* panel indicates a non-specific PCR product (dimers of primers). **C.** RT-qPCR quantification of the steady-state expression of canonical mRNAs corresponding to the tracRNAs validated in Fig. 1C. Two-tailed paired t-test (* *P*-val<0.05 ; ** *P*-val<0.01). **D.** Western-blot showing the validation of *DDX17* silencing.

Supp. Figure 3. The expression of the *DDX5* gene is reduced in high risk neuroblastomas. **A.** Kaplan-Meier curves showing the overall survival of neuroblastoma patients, separated in 2 groups of high and low *DDX5* expression (relative to the median value of the group). Left diagram: all patients (n=498). Right diagram: stage 4 patients (n=183). The number of patients in each group and for each time point is indicated below. **B.** Box plot of *DDX5* expression relative to tumor stage (INSS classification). ANOVA corrected for multiple comparisons with Tukey's tests. Only the comparison between Stage 4 and other stages is shown, the other comparisons were not significant. **C.** Box plot of *DDX5* expression relative to low/high risk classification. Two-tailed *t*-test. **D.** Box plot of *DDX5* expression relative to amplified (A) or non amplied (NA) *MYCN* status. Two-tailed *t*-test.

Supp. Figure 4. A. RT-qPCR quantification of the steady-state expression of canonical mRNAs corresponding to the tracRNAs and readthrough transcripts analysed in Fig. 3B, upon MYCN overexpression. Ratio paired *t*-test (*P-val<0.05 ; **P-val<0.01 ; ***P-val<0.001). **B.** RT-qPCR quantification of the steady-state expression of canonical mRNAs corresponding to the tracRNAs and

readthrough transcripts analysed in Fig. 3D, upon *MYCN* silencing. Unpaired Mann-Whitney test (*P-val<0.05 ; **P-val<0.01).

Supp. Figure 5. ChIP-qPCR analysis (n=2) of dCas9-MYCN and dCas9-GFP binding near the 3' end of *MAPK7*. ChIP data were normalised to their respective control (ChIP with IgG) and expressed as the relative enrichment to a negative region (*ADCK4* gene).

Legends for Supplementary Tables

Supp. Table 1. List of the 282 tracRNAs whose expression is changed upon siDDX17/DDX5 treatment, identified by the Arriba pipeline. The 97 most significant tracRNAs have their *p*-value cell highlighted in green. The last 2 columns report the tracRNAs identified by Shi *et al* in neuroblastoma tumours, with their number of occurences and corresponding frequency. The ID of these tracRNAs is highlighted in red, and the 25 most significant are in yellow characters. The second tab of the table compiles the merged list of genes whose termination is affected by DDX17/DDX5 depletion, generating readthrough transcription (our previous work, Terrone *et al.*, 2022) and tracRNA formation (this study).

Supp. Table 2. List of primers and siRNAs used in this study.

Supp. Table 3. List of public ChIP-seq datasets used to analyse MYCN binding.
PMID 32286315: Upton K *et al.* (2020). Epigenomic profiling of neuroblastoma cell lines. Sci Data 7(1):116. doi: 10.1038/s41597-020-0458-y.
PMID 29262328: Büchel *et al.* (2017). Association with Aurora-A Controls N-MYC-Dependent Promoter Escape and Pause Release of RNA Polymerase II during the Cell Cycle. Cell Rep 21(12):3483-3497. doi: 10.1016/j.celrep.2017.11.090.
PMID 29379199: Zeid R *et al.* (2018). Enhancer invasion shapes MYCN-dependent transcriptional amplification in neuroblastoma. Nat Genet 50(4):515-523. doi: 10.1038/s41588-018-0044-9.

Supp. Figure 1A

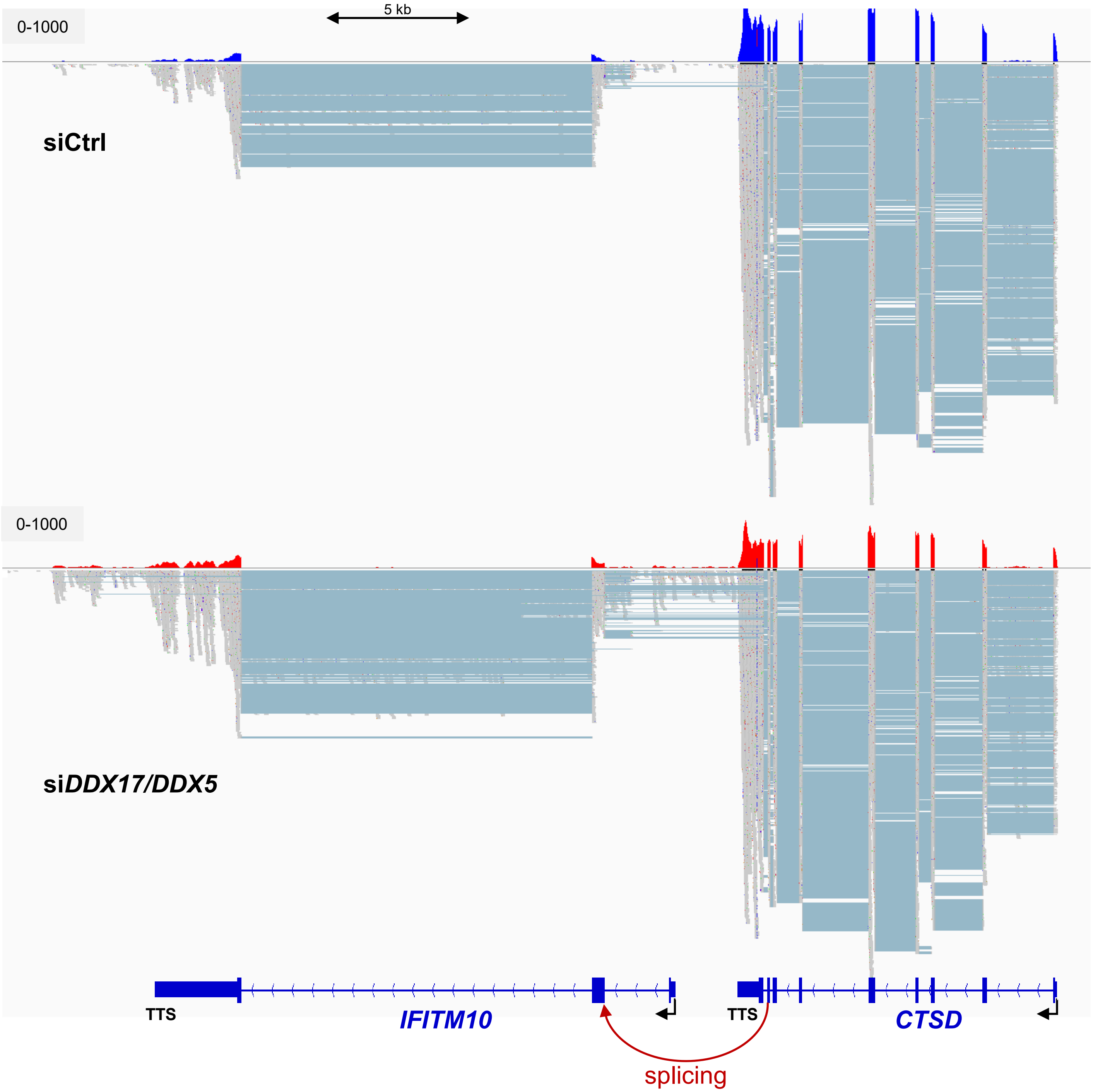

Supp. Figure 1B

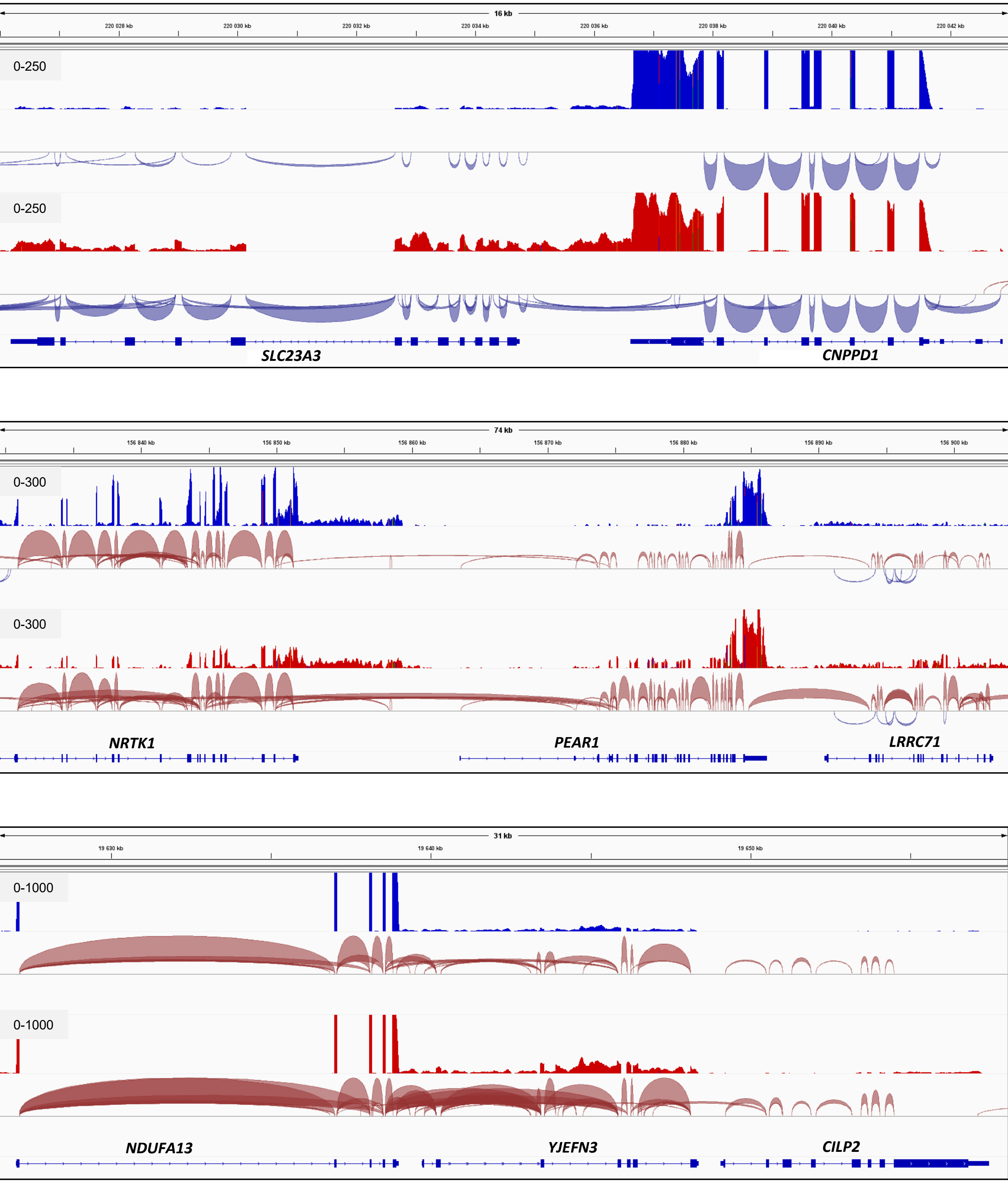

Supp. Figure 1B

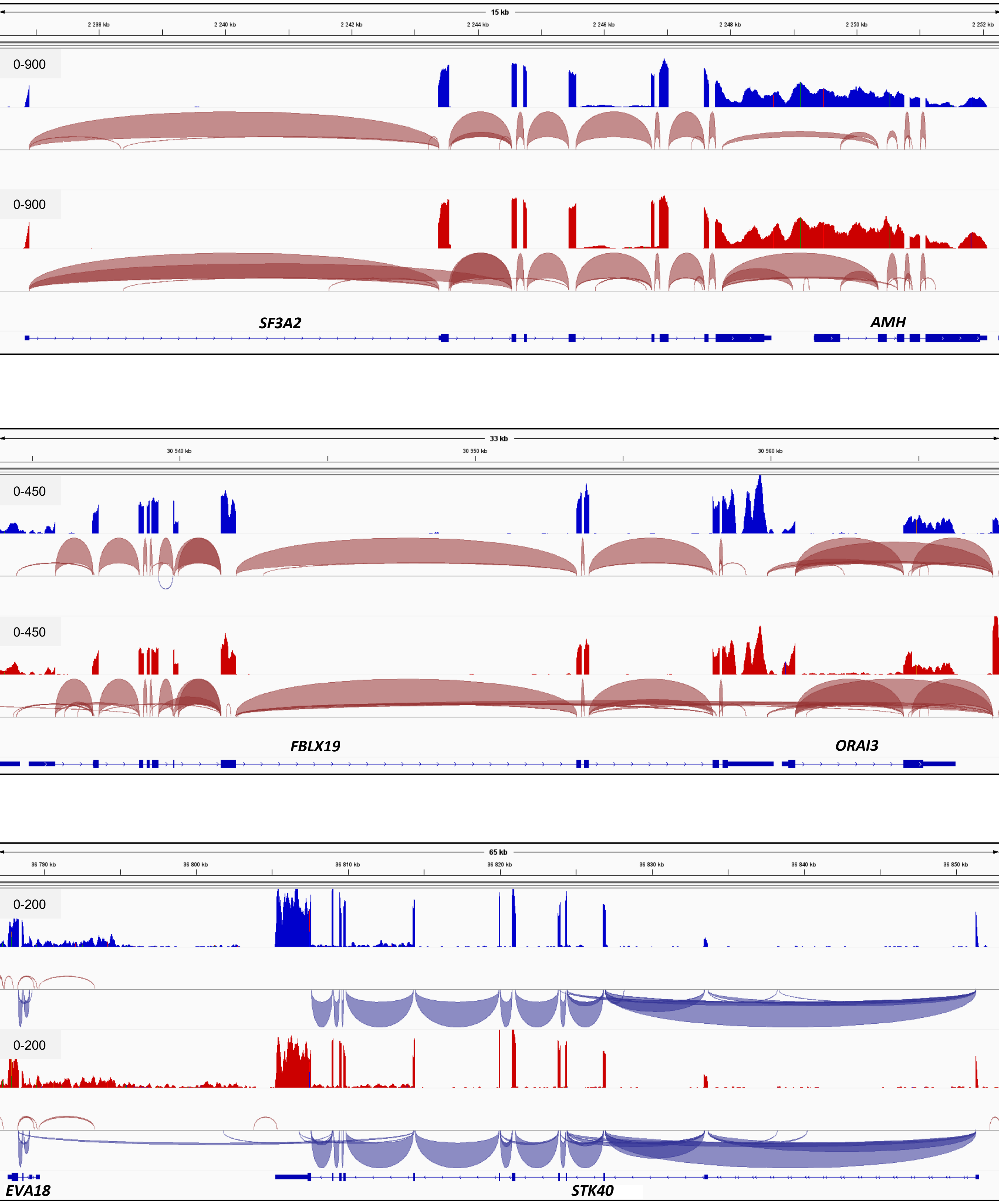

Supp. Figure 1B

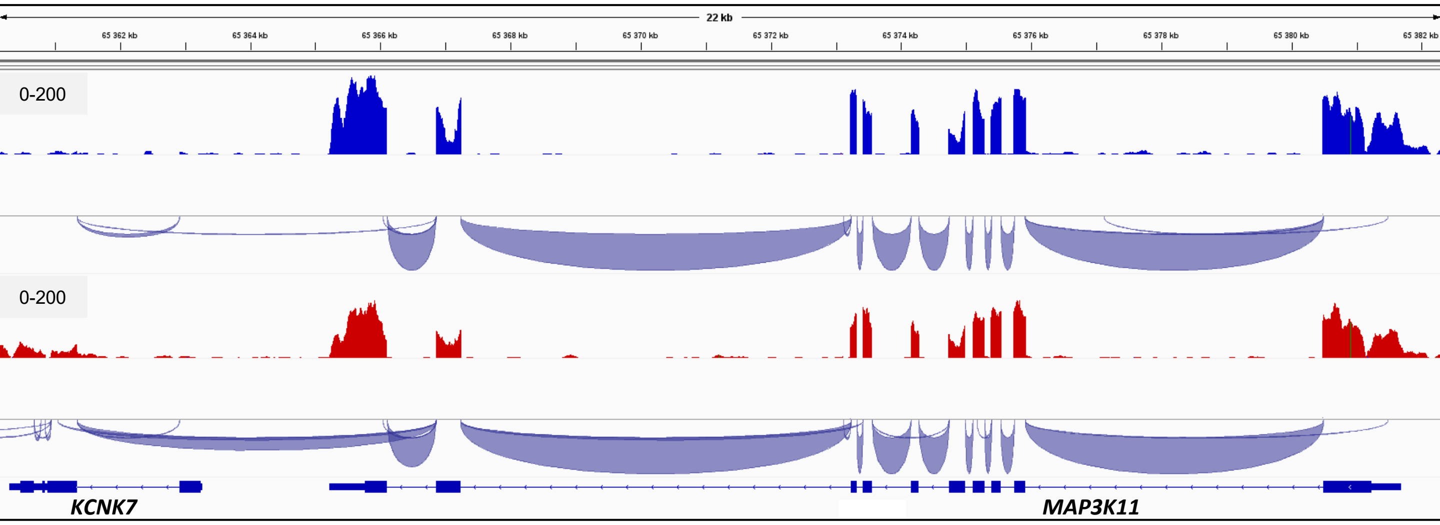

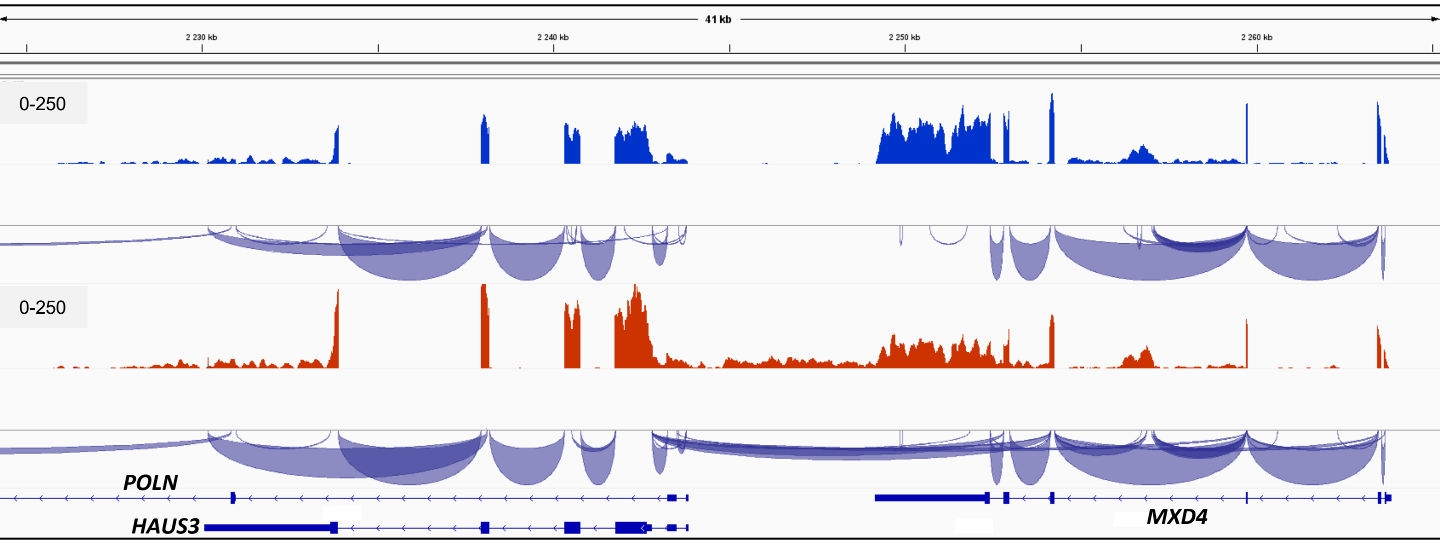

Supp. Figure 2

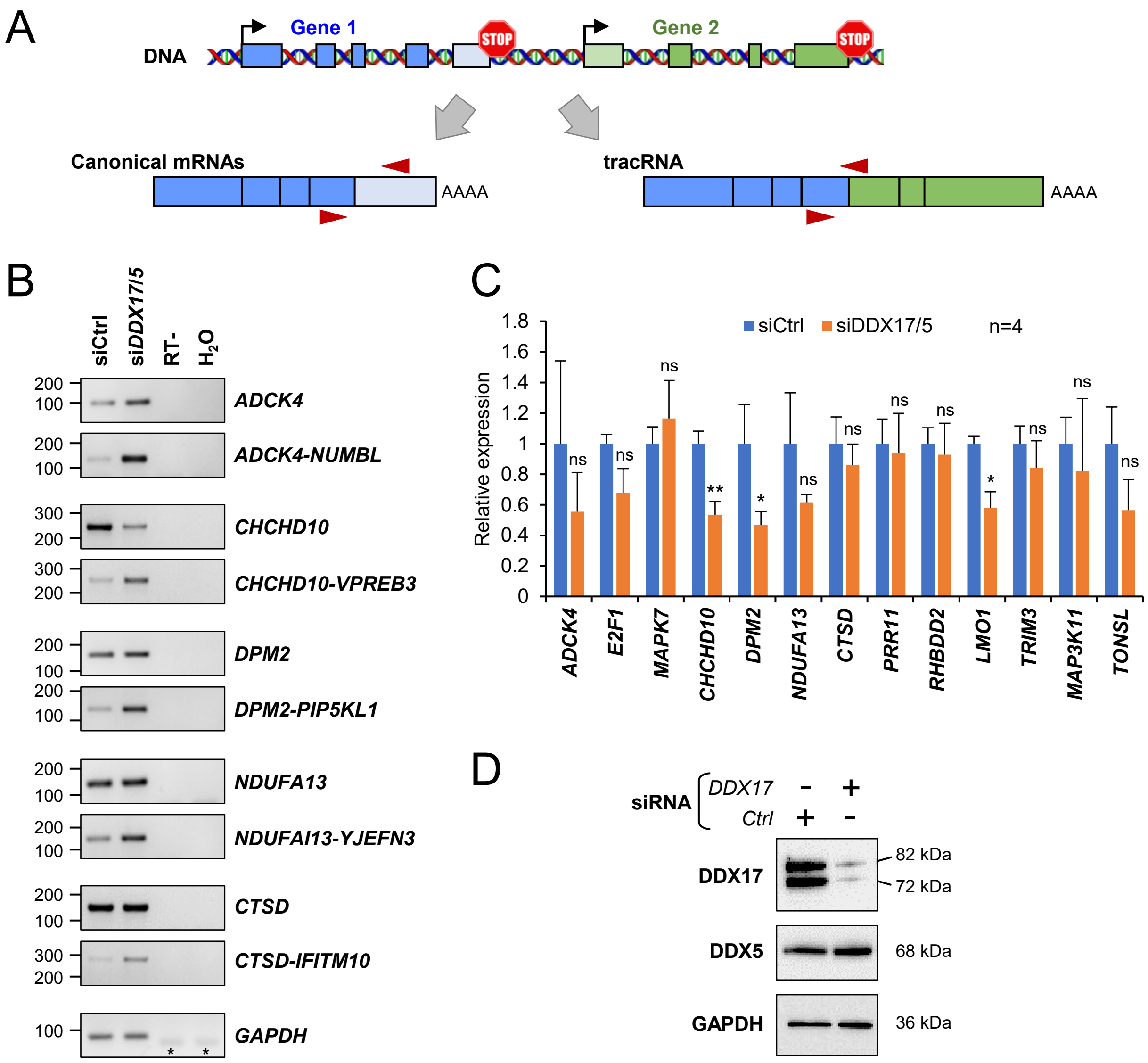

Supp. Figure 3

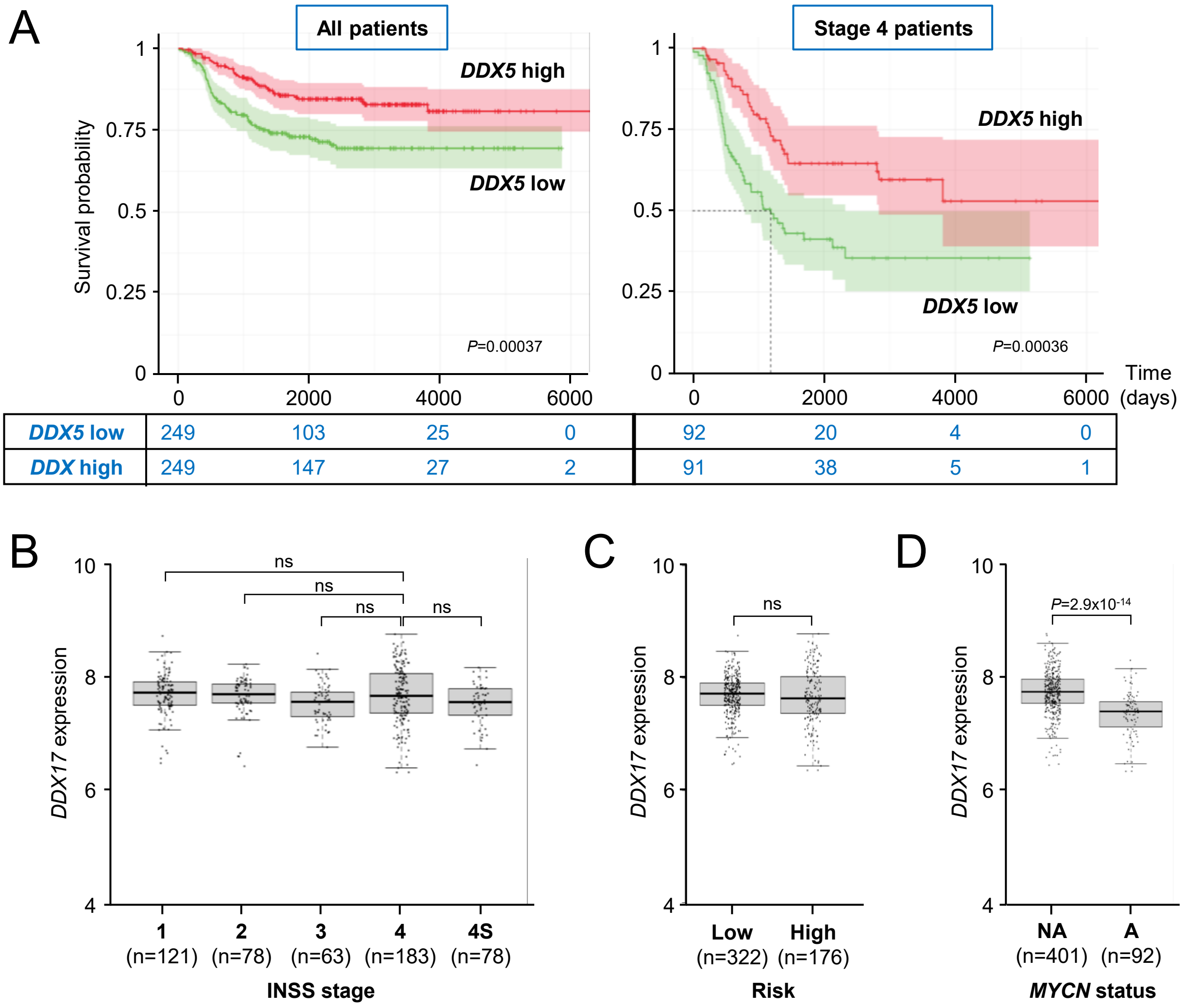

Supp. Figure 4

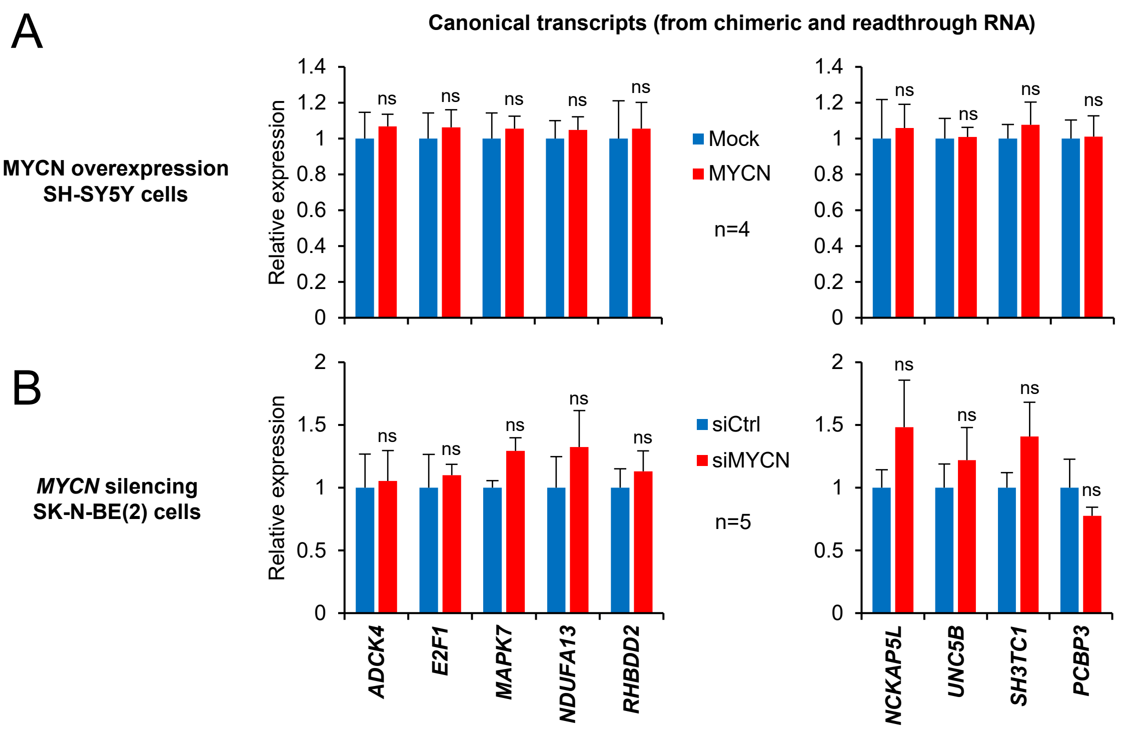

Supp. Figure 5

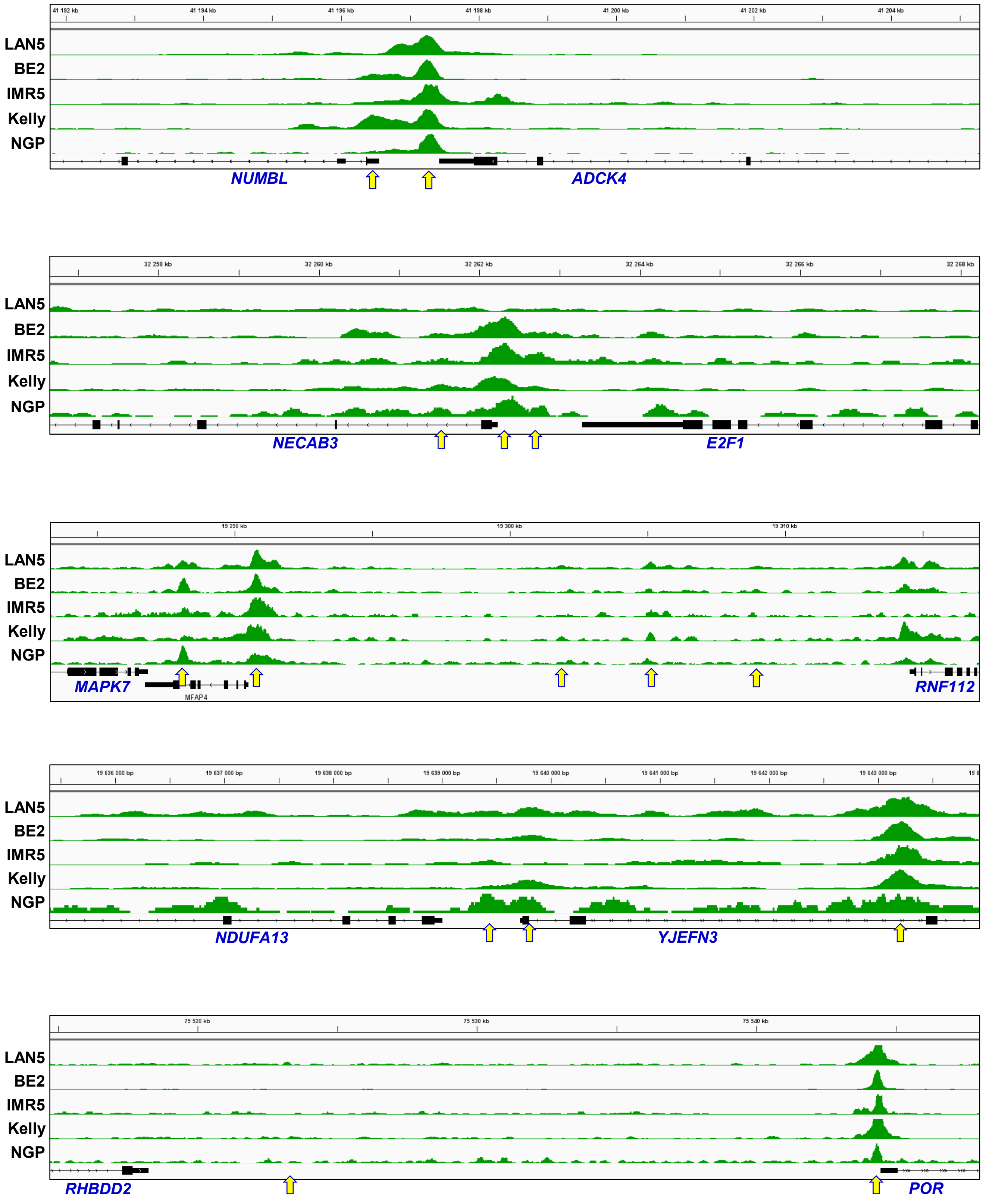

Supp. Figure 5

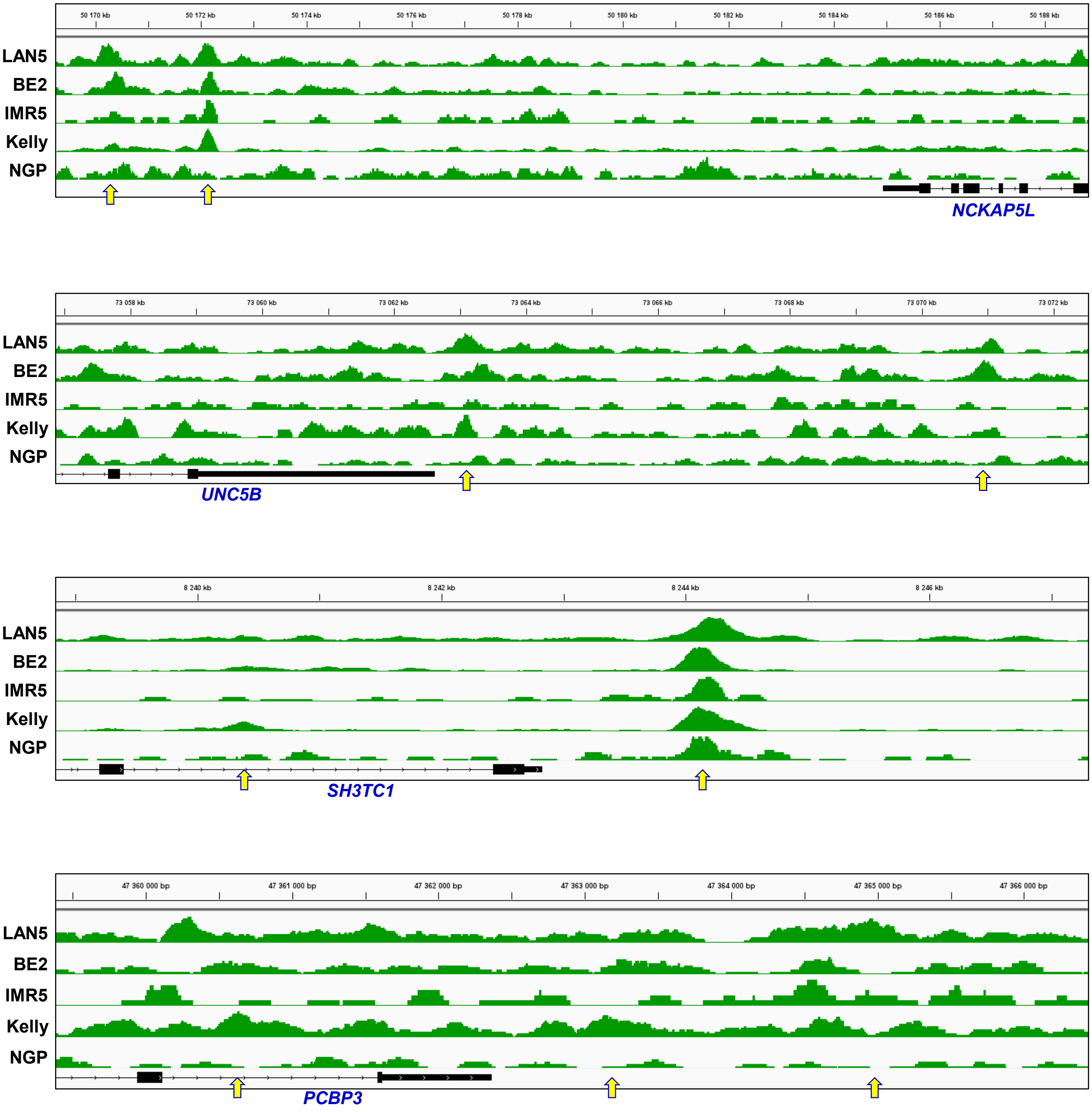

Supp. Figure 6

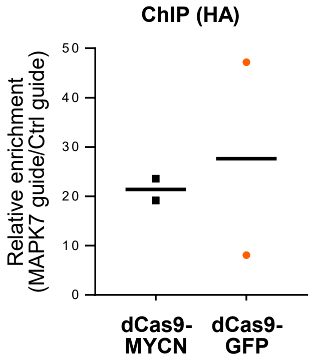

Supplement: Supplementary file 4 — Supplementary file4 (PDF 936 KB) [file 18_2026_6254_MOESM4_ESM.pdf]
